# Fast and Ultrasensitive Electrochemical Detection for Antiviral Drug Tenofovir Disoproxil Fumarate in Biological Matrices

**DOI:** 10.3390/bios12121123

**Published:** 2022-12-03

**Authors:** Jingyun Xiao, Shuting Shi, Liangyuan Yao, Jinxia Feng, Jinsong Zuo, Quanguo He

**Affiliations:** 1 School of Life Science and Chemistry, Hunan University of Technology, Zhuzhou 412007, China; 2 Hunan Qianjin Xiangjiang Pharmaceutical Joint Stock Co., Ltd., Zhuzhou 412001, China; 3 Geriatric Rehabilitation Department, Zhuzhou People’s Hospital, Zhuzhou 421007, China

**Keywords:** Tenofovir disoproxil fumarate, zirconium oxide, multiwalled carbon nanotubes, differential pulse voltammetry, biological samples

## Abstract

Tenofovir disoproxil fumarate (TDF) is an antiretroviral medication with significant curative effects, so its quantitative detection is important for human health. At present, there are few studies on the detection of TDF by electrochemical sensors. This work can be a supplement to the electrochemical detection of TDF. Moreover, bare electrodes are susceptible to pollution, and have high overvoltage and low sensitivity, so it is crucial to find a suitable electrode material. In this work, zirconium oxide (ZrO_2_) that has a certain selectivity to phosphoric acid groups was synthesized by a hydrothermal method with zirconyl chloride octahydrate as the precursor. A composite modified glassy carbon electrode for zirconium oxide-chitosan-multiwalled carbon nanotubes (ZrO_2_-CS-MWCNTs/GCE) was used for the first time to detect the TDF, and achieved rapid, sensitive detection of TDF with a detection limit of sub-micron content. The ZrO_2_-CS-MWCNTs composite was created using sonication of a mixture of ZrO_2_ and CS-MWCNTs solution. The composite was characterized using scanning electron microscopy (SEM) and cyclic voltammetry (CV). Electrochemical analysis was performed using differential pulse voltammetry (DPV). Compared with single-material electrodes, the ZrO_2_-CS-MWCNTs/GCE significantly improves the electrochemical sensing of TDF due to the synergistic effect of the composite. Under optimal conditions, the proposed method has achieved good results in linear range (0.3~30 μM; 30~100 μM) and detection limit (0.0625 μM). Moreover, the sensor has the merits of simple preparation, good reproducibility and good repeatability. The ZrO_2_-CS-MWCNTs/GCE has been applied to the determination of TDF in serum and urine, and it may be helpful for potential applications of other substances with similar structures.

## 1. Introduction

Acquired Immune Deficiency Syndrome (AIDS) and hepatitis B can cause a series of physical and mental health problems, about which the global community has become increasingly concerned. AIDS is caused by human immunodeficiency virus (HIV), which mainly causes damage to CD4 T cells [1], affecting the human immune system and causing the human body to become infected with other diseases or even die, due to the loss of resistance to diseases. Hepatitis B is one of the most dangerous forms of hepatitis. It is a disease caused by hepatitis B virus (HBV) and can cause damage to liver tissue [2]. Antiretroviral drugs are used in the treatment of retroviral infection. They can effectively treat HIV and HBV, and according to their molecular mechanism and drug resistance distribution, they are divided into six types: nucleoside reverse transcriptase inhibitors (NRTIs), non-nucleoside reverse transcriptase inhibitors (NNRTIs), integrase inhibitors, protease inhibitors (PIs), fusion inhibitors, and coreceptor antagonists [3]. One of the NRTIs, Tenofovir disoproxil fumarate (TDF, trade name Viread^®^) was researched and developed by Gilead Sciences, Inc. Its International Union of Pure and Applied Chemistry (IUPAC) name is Bis{[(isopropoxycarbonyl)oxy]methyl}({[(2R)-1-(6-amino-9H-purin-9-yl)-2-propanyl]oxy}methyl) phosphonate fumarate. TDF has been approved by the Food and Drug Administration (FDA) for the treatment of AIDS and hepatitis B, and it appears in the list of essential drugs of the World Health Organization (WHO) [4]. TDF is a precursor of Tenofovir (TFV). The oral bioavailability of TFV is low, while that of TDF is greatly improved [5]. At the same time, compared with other mainstream NRTIs, TDF has the advantages of reducing the rate of development of drug resistance, longer half-life and fewer side effects [6]. The possibility of drug resistance of TDF is very low [7]. These characteristics make TDF more popular in the treatment of HIV and HBV. After oral administration of TDF, it is first metabolized into TFV in vivo. Next, this is phosphorylated into the active metabolite Tenofovir bisphosphate under the action of cell kinase. After this, it actively inhibits virus activity, thus acting as a treatment. After 3 days of administration, TDF is excreted in vivo through glomerular filtration and the active tubular transport system, such that about 70%~80% of it is excreted in urine. At the same time, TDF does not need to be taken on an empty stomach; on the contrary, it can have improved bioavailability when it is consumed with high-fat food [8]. However, long-term use of TDF may lead to nephrotoxicity [9] and halisteresis [10]. Therefore, it is of great significance for individualized drug delivery, rational drug use, and precision medicine to quantitatively detect the content of TDF.

Nowadays, several analytical techniques for detection TDF have been reported upon, including spectrophotography [11,12,13], ultraviolet spectrophotometry [14,15], liquid chromatography tandem mass spectrometry [16], reversed-phase high-performance liquid chromatography [17], ultra-high efficiency supercritical fluid chromatography [18], high-performance thin layer chromatography [19], and so on. Although these traditional methods have been widely used and these technologies are mature, they often need large-scale precision instruments, and the pretreatment process is often complex. In this case, electrochemical methods are being gradually developed. Electrochemical analysis is used to determine the different oxidation states of an element in solution. This technique is more advantageous for analyzing the active components of drugs with extremely low detection limits. It also allows the determination of the mechanisms of a given drug, through the study of its electrochemical behavior. This method is not affected by excipients. This means that electrochemical detection can be directly used to detect drugs, with the advantages of speed, high sensitivity, low cost, and miniaturization [20,21]. In addition to modifying the electrode to detect substances [22], electrochemistry is also used in photocatalysis [23], disease screening and diagnosis [24,25], real-time monitoring of living cells [26], brain electrodes [27,28], and so on. At present, only one document on the use of a bare electrode has been reported on, for the electrochemical detection of TDF [29]. However, the bare electrode is easily polluted and interfered with, and has the disadvantages of high overvoltage and low sensitivity [30], so it is important to modify the electrode. Materials used for the surface modification of glassy carbon electrodes (GCEs) for electrochemical sensors mainly include carbon-based nanomaterials (such as carbon nanotubes and graphene), metal nanoparticles, metal oxide nanoparticles, and polymer materials. The materials used for electrochemical sensors should have good conductivity and selectivity to given drug molecules [31]. Therefore, it is necessary to choose suitable electrode materials to design a sensitive, cheap and effective electrochemical method, to determine TDF.

Metal oxide nanostructures are an ideal class of sensing materials [32,33,34,35,36,37], because of their large surface-to-volume ratio, high electroactive surface area, and good electrical conductivity [38]. These characteristics lead to their widespread use in electrochemical detection. They are usually used in combination with conductive support materials, to improve their electrochemical properties. At the same time, metal oxides have the advantages of rich sources and low cost. They have been extensively used in the preparation of electrochemical sensors and successfully used to analyze a variety of substances [23,39,40,41,42,43,44,45,46,47], such as biological small molecules, food additives, pesticides [48] and drugs. Zirconium oxide (ZrO_2_) is an inorganic oxide crystal with thermal stability, chemical inertia and avirulence. Additionally, it has a strong affinity for the phosphonic part. It was originally studied for use in the detection of enriched phosphopeptides, captured phosphoprotein and organophosphorus pesticides [49]. It has potential applications in catalysis, solid oxide fuel cells, sensors and nuclear radiation shielding materials [50,51]. At present, the methods of synthesizing zirconium oxide include the sol–gel method, precipitation method, solvothermal synthesis, hydrothermal synthesis, etc. [52]. The synthesized pure zirconium oxide has three crystal forms: monoclinic phase (m-ZrO_2_), tetragonal phase (t-ZrO_2_) and cubic phase (c-ZrO_2_) [53]. Zirconium oxide has been used in an electrochemical sensor and biosensor [54]. ReddyPrasad et al. added zirconyl chloride octahydrate (ZrOCl_2_) and C-dots to chitosan solution, obtained a C-dots/ZrO_2_ nanocomposite modified electrode by ultrasound and electrodeposition, and successfully applied it in the detection of Parathion in rice samples [49]. Chen et al. prepared an alveolated zirconium oxide with a chitosan modified electrode for electrochemical detection of the antituberculosis drug Rifampicin, and achieved good results. It has been applied to biological samples such as human serum and urine [55]. Tan and Wu used zirconium chloride and alcohol to synthesize zirconium oxide for an oxygen molecular sensor and achieved good results [56]. It can be seen that zirconium oxide can be used as electrode modification material.

Multiwalled carbon nanotubes (MWCNTs) have the properties of large specific surface area, strong adsorption capacity, strong conductivity and strong catalytic properties [57], and they can be used to construct high-performance sensors. Although carbon materials have many advantages, they lack selectivity to target molecules when used alone in sensing devices [58]. In practical applications, MWCNTs are often combined with other materials to enhance selectivity. Chitosan (CS) is deacetylated from natural chitin. It not only has hydrophilic and hydrophobic groups, but also -NH_2_ and -OH groups. It has been reported that a chitosan modified electrode can be used to detect various substances [2,57,59], and it can promote the even and steady dispersion of MWCNTs [2]. Drawing on the idea of using composites, metal and carbon matrix composites are developed, in order to obtain good selectivity and excellent thermal and electrical properties. Therefore, in this work, zirconium oxide-chitosan-multiwalled carbon nanotubes composites were prepared to detect TDF.

A ZrO_2_-CS-MWCNTs mixture was evenly modified onto the smooth surface of a glassy carbon electrode (GCE) using the drop coating method, to get a modified electrode (ZrO_2_-CS-MWCNTs/GCE). The electrochemical properties of the ZrO2-CS-MWCNTs/GCE were studied using cyclic voltammetry (CV) and differential pulse voltammetry (DPV). The sensor shows good selectivity, repeatability, reproducibility and low detection limit. In addition, the prepared electrochemical sensor was used to detect chemicals in serum and urine, with satisfactory results.

## 2. Materials and Methods

### 2.1. Materials

Tenofovir disoproxil fumarate was provided by Hunan Qianjin Xiangjiang Pharmaceutical Joint Stock Co., Ltd. (Zhuzhou, China); zirconium oxychloride octahydrate, MWCNTs, and sodium acetate trihydrate were purchased from Aladdin Biochemical Technology Co., Ltd. (Shanghai, China); chitosan, sodium hydroxide, glycerol and glacial acetic acid were purchased from Sinopharm Chemical Reagent Co., Ltd. (Shanghai, China). Other reagents used in the laboratory were analytically pure and can be used directly, without purification. All solutions were prepared with deionized water (resistivity is 18.2 MΩ·cm).

The preparation method was to weigh 0.0636 g of TDF and dissolve it in deionized water, then dilute it with water in a 100 mL brown volumetric flask to prepare a stock solution, and keep it away from light. A solution of 1 M acetic acid and 1 M sodium acetate solution was mixed to prepare an acetic acid-sodium acetate buffer as the supporting solution of the working solution.

### 2.2. Apparatus

Every electrochemical measurement was performed using a CHI660E electrochemical workstation (Shanghai Chenhua Instruments Co., Ltd., Shanghai, China). The pH of the solution was measured using a digital PHS-3C pH meter (Shanghai Leici Instrument Factory, Shanghai, China). The morphologies of nano-materials were characterized using a scanning electron microscope (SEM, ZEISS Sigma 300, Jena, Germany) and an energy dispersion spectrometer (EDS) attached to the SEM. The crystal structures of the nanomaterials were characterized using X-ray diffraction (XRD, Rigaku Smart Lab SE, Tokyo, Japan).

### 2.3. Preparation of ZrO_2_

ZrO_2_ was synthesized using a hydrothermal method. 1.289 g zirconium oxychloride octahydrate was turned into a 4 mM zirconium oxychloride octahydrate solution with deionized water, and then added to the 8 mM glycerol solution. At this time, their molar ratio was 1:2. Then they were mixed evenly with a magnetic stirrer, and 0.2 M sodium hydroxide solution was added to adjust the solution to pH = 9. When the solution presented as a white emulsion, the mixture was transferred to a 100 mL stainless steel autoclave and reacted at 180 °C for 18 h, after which it was cooled at room temperature. The obtained white precipitate was centrifuged and washed with water and ethanol multiple times, and finally dried in a vacuum at 60 °C for 3 h to obtain zirconium oxide nanoparticles.

### 2.4. Preparation of ZrO_2_-CS-MWCNTs Composite Dispersion

To prepare the composite dispersion, 1 mg ZrO_2_ and 1 mg MWCNTs were scattered in 2 mL acetic acid solution (2% *v*/*v*) containing 1 mg CS. Ultrasonication was performed for 1 h to obtain a uniform dispersion. Used as a dispersant, 2% acetic acid can easily dissolve CS.

### 2.5. Fabrication of Modified Electrode

Firstly, the bare electrode was thoroughly polished on suede with 1.0, 0.3 and 0.05 μM aluminum oxide (Al_2_O_3_) slurries in that order. After each polishing process, the electrode was washed with ethanol and deionized water in turn, until a mirror-like surface was formed. Finally, the GCE was dried under an infrared lamp. In order to prepare the TDF electrochemical sensor (ZrO_2_-CS-MWCNTs/GCE), ZrO_2_-CS-MWCNTs composite dispersion was carefully dropped on the electrode surface. After the electrode was dried under an infrared lamp, the sensor we prepared was obtained. The fabrication process of the modified electrode is displayed in Figure S1. Similarly, ZrO_2_/GCE and CS-MWCNTs/GCE were also fabricated in accordance with the above process.

### 2.6. Electrochemical Detection of TDF

All electrochemical measurements in this work were carried out on a CHI660E electrochemical workstation including a conventional three-electrode system. ZrO_2_-CS-MWCNTs/GCE was used as the working electrode, a platinum wire electrode was used as the auxiliary electrode, and a saturated calomel electrode (SCE) was used as the reference electrode in this system. Cyclic voltammetry (CV) was used for characterization in a solution of 5 mM potassium ferricyanide, 5 mM potassium ferrocyanide and 0.1 M potassium chloride. The performance, optimization of measurement conditions, selectivity, repeatability, reproducibility and stability of TDF on ZrO_2_-CS-MWCNTs/GCE were tested using differential pulse voltammetry (DPV). Before each experiment, the newly prepared electrode needed to be activated and have dust removed by scanning 20 cycles using CV in 1 M acetic acid–sodium acetate buffer solution. Next, the treated electrode was inserted into the working solution of TDF that had been diluted with acetic acid-sodium acetate buffer solution, and stirred continuously for 90 s at the potential of 0.2 V. Then the TDF was measured by the DPV at the scanning rate of 0.1 V/s within the potential range of 1.0 V~1.5 V. For DPV, the potential pulse amplitude is 0.05 V, potential pulse width is 0.05 s, data sampling width is 0.0167 s, and potential pulse period is 0.2 s. After every measurement, stirring and using the electrochemical workstation were suspended. After this, under the condition of constant potential of −1 V, the corresponding potential of 30 s was applied in the detection solution to prevent electrode passivation. Each electrochemical measurement was carried out at room temperature.

### 2.7. Sample Preparation

In this experiment, serum and urine were used as the actual samples. The blood of a drug-free human was acquired from the local hospital, and the urine was from the laboratory staff. First, 1 mL of blood and an appropriate amount of TDF stock solution were centrifuged for 30 min with 4000 rpm at room temperature. Next, 0.9 mL of 15% (*w*/*v*) zinc sulfate solution–acetonitrile (50/40, *v*/*v*) was added to remove protein from the blood sample [60]. The supernatant was collected, and then acetic acid-sodium acetate buffer was added to make the supernatant and buffer reach a constant volume of 10 mL to prepare the serum sample containing TDF of the required concentration. Additionally, 1 mL of urine and an appropriate amount of TDF stock solution were centrifuged for 30 min with 4000 rpm. The collected supernatant was diluted to 10 mL with acetic acid-sodium acetate buffer to prepare a urine sample containing TDF of the required concentration. Both were detected using the standard addition method.

## 3. Results and Discussion

### 3.1. Crystal Phase and Morphology Characterization

**Figure 1:**
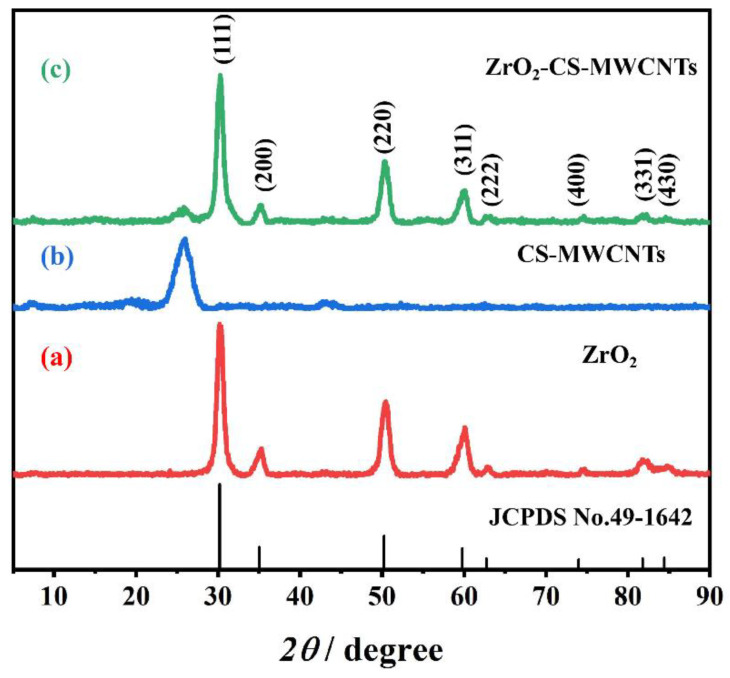
The X-ray diffraction (XRD) patterns of ZrO_2_ (**a**), CS-MWCNTs (**b**) and ZrO_2_-CS-MWCNTs composites (**c**).

The crystal structures of the ZrO_2_, CS-MWCNTs and ZrO_2_-CS-MWCNTs composites were studied using XRD. As shown in Figure 1, CS-MWCNTs showed an obvious peak at 2*θ* = 26°, which was consistent with the reported typical diffraction peaks of MWCNTs [61]. It can also clearly be seen from Figure 1 that the diffraction peaks appeared at 2*θ* = 30.12°, 34.96°, 50.22°, 59.74°, 62.68°, 73.94°, 81.76° and 84.40°, which corresponded to the (111), (200), (220), (311), (222), (400), (331) and (430) crystal planes of ZrO_2_. These peak positions were compared with the diffraction peak of a c-ZrO_2_ standard card (JCPDS, No. 49-1642), which indicated that the ZrO_2_ synthesized by us was of cubic phase structure. In the XRD pattern of the ZrO_2_-CS-MWCNTs composite, not only the characteristic diffraction peak of CS-MWCNTs, but also the characteristic diffraction peak of ZrO_2_ can be observed; this was the first indication that the composite had been successfully prepared.

**Figure 2:**
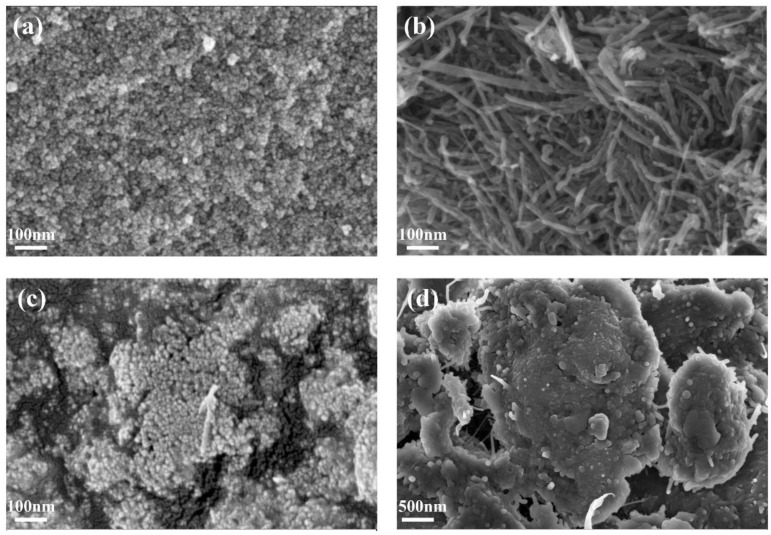
Scanning electron microscope (SEM) images of the ZrO_2_ (**a**), CS-MWCNTs (**b**) and ZrO_2_-CS-MWCNTs composites (**c**,**d**).

The morphologies of the ZrO_2_, CS-MWCNTs and ZrO_2_-CS-MWCNTs composites were characterized using SEM, as shown in Figure 2. It can be observed in Figure 2a that ZrO_2_ had a small spherical shape with relatively uniform particles. In Figure 2b, CS-MWCNTs showed tubular structure. Figure 2c,d illustrates the morphologies of ZrO_2_-CS-MWCNTs composites at different magnification. These SEM images showed that a dense covering of spherical ZrO_2_ was present on the surface of CS-MWCNTs; this phenomenon may be caused by the particle size difference between the them. At the same time, they form a honeycomb shape, which is conducive to the attachment of the target detection object.

Figure S2a shows the EDS of the ZrO_2_-CS-MWCNTs composites. The peaks of carbon (C), nitrogen (N), oxygen (O) and zirconium (Zr) can be observed in EDS. Their contents in the composite were 54.27%, 5.59%, 15.62% and 24.52%, respectively, and their atomic ratios were 73.32%, 6.48%, 15.84% and 4.36%, respectively. The mass ratio of oxygen to zirconium in the composite was about 0.64:1, which is greater than the theoretical ratio of zirconium dioxide 0.35:1, and the atomic ratio is about 3.6:1, which is greater than the theoretical ratio of zirconium dioxide of 2:1. This may be due to the presence of oxygen in chitosan and air. Combined with the XRD analysis of the composites, the results further confirmed that ZrO_2_-CS-MWCNTs composites had been successfully prepared. Figure S2c–f shows the element mapping of the designated area of ZrO_2_-CS-MWCNTs composites (Figure S2b), which more clearly shows the distribution of carbon, nitrogen, oxygen and zirconium in the ZrO_2_-CS-MWCNTs composites. It was found that they are evenly distributed in the composites, further indicating that the composite has been successfully prepared.

### 3.2. Electrochemical Characterization

**Figure 3:**
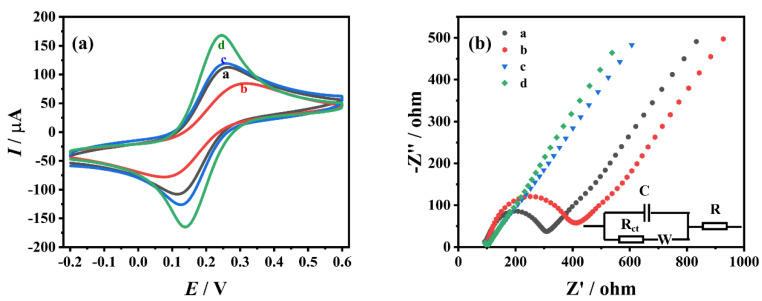
Cyclic voltammetry (CV) diagrams (**a**) and Nyquist plots (**b**) obtained on different electrodes in a solution of 5 mM K_3_[Fe(CN)_6_]^3−/4−^ and 0.1 M KCl (a: GCE, b: ZrO_2_/GCE, c: CS−MWCNTs/GCE, d: ZrO_2_−CS−MWCNTs/GCE).

GCE, ZrO_2_/GCE, CS-MWCNTs/GCE and ZrO_2_-CS-MWCNTs/GCE were immersed in a potassium ferricyanide–potassium ferrocyanide solution (5 mM), containing 0.1 M potassium chloride, and CV characterization was performed at a scanning speed of 0.1 V/s in the potential range of −0.2~0.6 V (Figure 3a). The current of oxidation peak (*I*_pa_) and reduction peak (*I*_pc_) on GCE was 108.4 μA and 109.9 μA, respectively. On ZrO_2_/GCE, the redox peaks of [Fe(CN)_6_]^3−/4−^ were slightly reduced; these were 69.32 μA and 69.07 μA, respectively. This may be due to the thin conductivity of ZrO_2_. On CS-MWCNTs/GCE, the currents of *I*_pa_ and *I*_pc_ were 113.8 μA and 125.6 μA, respectively. The values of these currents are slightly higher than those of GCE, due to the excellent conductivity and catalytic performance of multiwalled carbon nanotubes. Compared with other electrodes, ZrO_2_-CS-MWCNTs/GCE showed the largest redox peaks; the values of these currents were 177.3 μA and 172.1 μA, respectively. This may be due to the synergistic effect of ZrO_2_ and multiwalled carbon nanotubes, which improves the performance of the sensor. The surface active areas of these electrodes are calculated according to the Randles–Ševčík formula [62]:

(1) $$I_{p}=\left(2.69\times 10^{5}\right)n^{3/2}D^{1/2}v^{1/2}AC$$

In the Randles–Ševčík formula, *I*_p_ is the peak current of K_3_[Fe(CN)_6_] (A), *n* is the number of electrons transmitted, *A* is the surface active area (cm^2^), D is the diffusion coefficient of K_3_[Fe(CN)_6_] (7.6 × 10^−6^ cm^2^s^−1^), *v* is the scanning rate (V/s), and *C* is the concentration of K_3_[Fe(CN)_6_] (mol/cm^3^). The calculated effective areas of GCE, ZrO_2_/GCE, CS-MWCNTs/GCE and ZrO_2_-CS-MWCNTs/GCE are 0.092 cm^2^, 0.059 cm^2^, 0.097 cm^2^ and 0.151 cm^2^, respectively. The results show that the ZrO_2_-CS-MWCNTs composites notably improved the effective area value of the glassy carbon electrode.

Electrochemical impedance spectroscopy (EIS) is an effective method to characterize electronic heterogeneity at the solution/electrode interface. Figure 3b shows the Nyquist diagrams of GCE, ZrO_2_/GCE, CS-MWCNTs/GCE and ZrO_2_-CS-MWCNTs/GCE in potassium ferricyanide–potassium ferrocyanide solution (5 mM) containing 0.1 M potassium chloride. The figure includes the semicircular part of the high-frequency region and the linear part of the low-frequency region. The electron transfer resistance (R_ct_) of the electrode is equal to the semicircle diameter of the high-frequency region of the Nyquist diagram. This resistance regulates the electron transport kinetics of the redox probe on the electrode surface. Two obvious semicircles can be seen in GCE and ZrO_2_/GCE, and the semicircle diameter of ZrO_2_/GCE is larger than that of GCE, indicating that the impedance of ZrO_2_/GCE is larger than that of GCE, which may be due to the poor conductivity of ZrO_2_. In CS-MWCNTs/GCE and ZrO_2_-CS-MWCNTs/GCE, it can be seen that the curves obtained in the high-frequency region are almost straight lines, which indicates that their impedance is significantly reduced. The R_ct_ values of GCE, ZrO_2_/GCE, CS-MWCNTs/GCE and ZrO_2_-CS-MWCNTs/GCE are 210.4 Ω, 294.0 Ω, 35.23 Ω and 19.32 Ω, respectively. The impedance of ZrO_2_-CS-MWCNTs/GCE is the lowest, indicating that the combination of ZrO_2_ and CS-MWCNTs has a synergistic effect and is more conducive to the electrochemical reaction. This result is consistent with the CV characterization results.

### 3.3. Electrochemical Behavior of TDF on Different Electrodes

The electrochemical behaviors of different electrodes in 10^−4^ M TDF solution (acetic acid–sodium acetate buffer solution at pH 4.5) were investigated by DPV, as shown in Figure S3. It was found that the peak current of TDF is the smallest on ZrO_2_/GCE, while it is the largest on ZrO_2_-CS-MWCNTs/GCE, at more than eight times that of TDF on the bare electrode. This shows that the synergistic effect of ZrO_2_ and CS-MWCNTs enhances the electrocatalysis effect, which increases the electrochemical response of TDF.

### 3.4. Effect of pH

**Figure 4:**
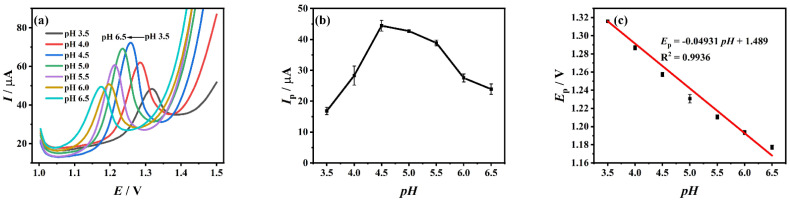
Differential pulse voltammetry (DPV) diagrams (**a**), current response (**b**) and the relationship diagram between peak potential and pH (**c**) of Tenofovir disoproxil fumarate (TDF) on ZrO_2_−CS−MWCNTs/GCE at different pH values.

The effect of the pH value of buffer solution on the peak current of 10^−4^ M TDF on ZrO_2_-CS-MWCNTs/GCE was studied using DPV. Figure 4a shows the corresponding DPV diagrams of 10^−4^ M TDF in the pH range of 3.5 to 6.5. It can be clearly seen that when the value of pH is 4.5, the maximum oxidation peak current of TDF can be obtained (Figure 4b), and when the pH value increases, the oxidation peak potential gradually moves negatively (Figure 4c), and the linear regression equation is: *E*_p_ (V) = −0.04931 *pH* + 1.489 (R^2^ = 0.9936). The oxidation reaction of TDF on the surface of ZrO_2_-CS-MWCNTs/GCE is a process with same number of protons and electrons because the slope of *E*_p_-*pH* is close to the theoretical value [63] of −0.0590 V/pH.

### 3.5. Effect of Scan Rate

**Figure 5:**
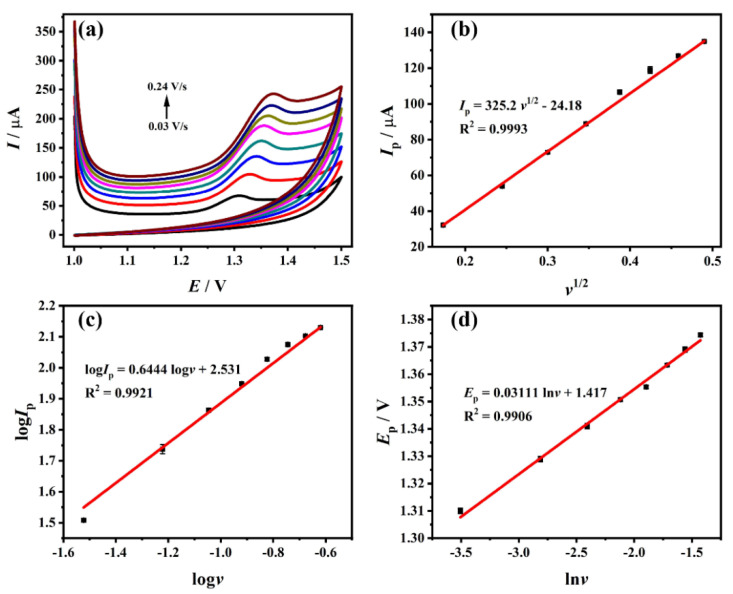
(**a**) CV diagrams of TDF on ZrO_2_−CS−MWCNTs/GCE at different scan rates (0.03 V/s ~ 0.24 V/s); (**b**) the plot of the peak current versus *v*^1/2^; (**c**) the plot of log*I*_p_ versus log*v*; (**d**) the plot of the peak potential versus ln*v*.

In this work, the effect of the scanning rate on the oxidation of 10^−4^ M TDF on the modified electrode was studied and compared using CV. Figure 5a shows the CV diagrams of TDF in a certain scanning rate range (0.03 V/s~0.24 V/s). It can be seen that the oxidation reaction of TDF on ZrO_2_-CS-MWCNTs/GCE is irreversible. As illustrated in Figure 5b,c, the linear regression equation of *v*^1/2^ and peak current can be expressed as: *I*_p_ (μA) = 325.2 *v*^1/2^ − 24.18 (R^2^ = 0.9993); the linear regression equation of log*v* and log*I*_p_ can be expressed as: log*I*_p_ = 0.6444 log*v* + 2.531 (R^2^ = 0.9921). The slope of log*v* and log*I*_p_ is 0.6444, between 0.5 and 1, and there is a linear relationship between *I*_p_ and *v*^1/2^, indicating that the oxidation reaction of TDF on ZrO_2_-CS-MWCNTs/GCE is a mixed control process, dominated by diffusion. Simultaneously, it can be concluded from Figure 5d that the peak potential of TDF is directly proportional to ln*v*, and the linear regression equation is expressed as: *E*_p_ (V) = 0.03111 ln*v* + 1.417 (R^2^ = 0.9906). In accordance with the Laviron equation [64]:

(2) $$E_{p}=E^{0}+\left(RT/\alpha nF\right)\ln\left(RTk^{0}/\alpha nF\right)+\left(RT/\alpha nF\right)\ln v$$

For the Laviron equation, *E*_p_, *E*^0^, *v*, *α*, *k*^0^, *n, T*, *F* and *R* respectively represent the peak potential (V), the formal potential (V), the scanning rate (V/s), the charge transfer coefficient, the standard heterogeneous velocity rate constant, the number of electron transfers, temperature in Kelvin (K), the Faraday constant (96,480 C/mol) and the molar gas constant (8.314 J/mol·K). Because the reaction of TDF on ZrO_2_-CS-MWCNTs/GCE is irreversible, it can be known that *α* = 0.5 [55]. It is calculated that *n* = 1.652, so TDF is an oxidation reaction involving two electrons on ZrO_2_-CS-MWCNTs/GCE. Combined with the conclusion obtained from the pH, it can be seen that the electrochemical oxidation of TDF on the electrode is a two-electron and two-proton transfer process. According to the existing reports on the oxidation mechanism of TFV (of which TDF is a precursor) [65,66], it can be speculated that the oxidation mechanism of TDF on ZrO_2_-CS-MWCNTs/GCE is as shown in Figure S4.

### 3.6. Effects of Deposition Conditions and Dropping Amount of ZrO_2_-CS-MWCNTs Composites

The effects of deposition time, deposition potential, and dropping amount of ZrO_2_-CS-MWCNTs composites on the electrochemical activity of 10^−4^ M TDF on ZrO_2_-CS-MWCNTs/GCE were analyzed using DPV. As shown in Figure S5a, the peak current first increases and then decreases with the increase of deposition time from 30~210 s, and reaches the maximum at 90 s. This may be because the supersaturated adsorption of TDF on the electrode surface leads to the passivation of ZrO_2_-CS-MWCNTs/GCE, which hinders the transfer of electrons and reduces the activity of the electrode. Therefore, 90 s is selected as the deposition time. As shown in Figure S5b, the deposition potential is between 0.0~1.0 V, the degree of TDF adsorbed on the electrode gradually increases, the peak current also first increases and then decreases, and reaches a maximum at 0.2 V. At this point, the TDF is fully adsorbed. With the growth of deposition potential, the background current also grows, which has a certain impact on the electrochemical response of TDF. Therefore, 0.2 V is selected as the optimum deposition potential. The film thickness of electrode modification material will also affect the determination of TDF. In the range of 1~9 μL, the effects of different dropping amounts of ZrO_2_-CS-MWCNTs composites on the TDF oxidation peak current were studied (Figure S5c). Between 1 and 5 μL, the peak current increases with the increase of the dropping amount. When the dropping amount exceeds 5 μL, the peak current decreases with further increases in dropping amount; this may be because the film thickness hinders the transmission of electrons. Therefore, 5 μL is selected as the best dropping amount. To sum up, the optimal detection conditions for the electrochemical detection of TDF with the modified electrode are a deposition time of 90 s, a deposition potential of 0.2 V, and a dropping amount of 5 μL.

### 3.7. Standard Curve and Detection Limit

**Figure 6:**
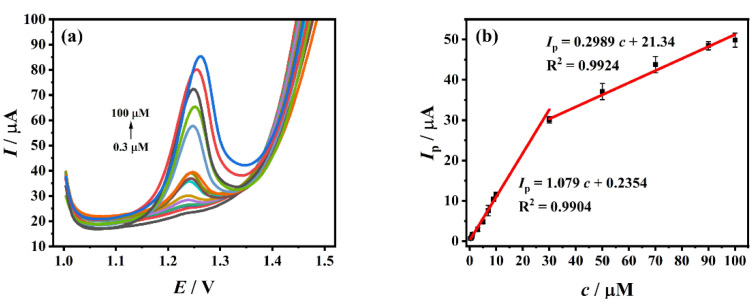
(**a**) DPV diagrams of different concentrations of TDF (0.3 μM~100 μM); (**b**) the linear relationship between the peak current and TDF concentration in the range of 0.3 μM~30 μM and 30 μM~100 μM.

Under the optimal detection conditions, DPV was used for the detection of different concentrations (0.3 μM~100 μM) of TDF, as shown in Figure 6a. Within this range, the oxidation peak current increases linearly with the increase of TDF concentration (Figure 6b, its specific data are in Table S1), and the linear regression equations are *I*_p_ (μA) = 1.079 *c* (μM) + 0.2354 (R^2^ = 0.9904) (0.3 μM~30 μM) and *I*_p_ (μA) = 0.2989 *c* (μM) + 21.34 (R^2^ = 0.9924) (30 μM~100 μM). The standard errors for estimated parameters of linear regressions for the peak current and TDF concentration can be calculated by the Formula (3):

(3) $$\sigma_{est}=\sqrt{\sum^{\;}{\left(Y-Y^{\prime }\right)}^{2}/\left(N-K\right)}\;$$

In Formula (3), *σ*_est_ is the standard error of the estimate, *Y* is an actual value, *Y’* is a predicted value, and *N* is the number of pairs of value, *K* is the number of parameters. These values of standard errors for estimated parameters of linear regressions for *I*_p_ and TDF concentration can be calculated to 0.9479 and 1.280 respectively. It can be seen that the approximation error between the estimator and its true value is smaller.

When the signal-to-noise ratio was 3 (S/N = 3), the limit of detection (LOD) was estimated using the Formula (4) [21]:

(4) LOD=3 s/m 

In Formula (4), s is the standard deviation of 5 blank signals and equals to 0.02248 μA, m is the slope of the calibration line within the corresponding range and equals to 1.079 μA/μM. The LOD is accordingly calculated as 0.0625 μM. The TDF electrochemical sensor prepared in this work is compared with other detection methods. The results are shown in Table 1. The detection limit is one of the important indicators to judge the sensitivity of a method. The lower the detection limit, the better the sensitivity of the method. Compared with other traditional methods, ZrO_2_-CS-MWCNTs/GCE, as an electrochemical method, is faster and more sensitive. Although spectrophotometry uses acid triphenylmethane dye as a material to make TDF complexed with dye ion pairs [11], the linear range and detection limit of the current work are still superior to this method. Compared with the existing electrochemical methods, although TDF has certain electrochemical activity that can be directly detected with bare electrodes, ZrO_2_-CS-MWCNTs/GCE overcomes the shortcomings of direct detection with bare electrodes, and shows a wider range of linearity and lower limit of detection. The results show that the sensor has good sensitivity.

### 3.8. Selectivity

In order to investigate the anti-interference of ZrO_2_-CS-MWCNTs/GCE, under the best conditions, potential interfering substances in body fluids and products were added to 10^−4^ M TDF solution. As shown in Figure S6, within ±5% of the allowable relative error, 100 times amounts of Fe^3+^, Cu^2+^, K^+^, Zn^2+^ and Na^+^ did not significantly interfere with the electrochemical response of TDF on ZrO_2_-CS-MWCNTs/GCE, and 10 times amounts of glucose, L-glutamic acid, dopamine hydrochloride, citric acid, uric acid and Fumaric acid also did not interfere with the electrochemical response of TDF. The results show that the sensor has good anti-interference properties for the analysis of TDF, which makes the detection of TDF in actual samples feasible. It can therefore be used for the detection and analysis of complex samples.

### 3.9. Repeatability, Reproducibility and Stability

The indicators for evaluating the reliability and accuracy of the electrode also include repeatability, reproducibility and stability. These indicators of ZrO_2_-CS-MWCNTs/GCE were studied through DPV in 10^−4^ M TDF working solution, which provided a basis for demonstrating the reliability and accuracy of this method. On a ZrO_2_-CS-MWCNTs/GCE, the peak currents were recorded for five consecutive times (Figure S7a). The relative standard deviation (RSD) of these measured peak currents is 4.94%, implying that the ZrO_2_-CS-MWCNTs/GCE has good repeatability in TDF determination. In addition, five different ZrO_2_-CS-MWCNTs/GCEs were obtained through the same preparation process to research the reproducibility. The peak currents of different electrodes under the same conditions are shown in Figure S7b. The RSD of these measured peak currents is 4.60%, which means that the ZrO_2_-CS-MWCNTs/GCE shows good reproducibility in TDF determination. Two ZrO_2_-CS-MWCNTs/GCEs were prepared in the same way and reused for five days. It was found that the peak currents of ZrO_2_-CS-MWCNTs/GCE remained 92.23% and 93.15%, respectively (Figure S7c), indicating that the modified electrode has good stability.

### 3.10. Sample Detection

Tenofovir plasmatic concentration has a high predictive value in monitoring the prevention of HIV infection by TDF, and different concentrations have different protection rates against HIV [68]. In addition, the monitoring of plasmatic and urinary drug concentrations of TDF has a certain reference value for the clinical treatment of CHB patients [69]. It can be seen that the treatment effect on HIV and CHB patients can be predicted by measuring the concentration of TDF in serum and urine. In order to study the feasibility of using ZrO_2_-CS-MWCNTs/GCEs to detect the content of TDF in actual situations, human serum and urine were taken as actual samples and tested by the method of standard addition and recovery. Under optimal conditions, the results are shown in Table 2 (specific urine and serum measurement data are shown in Table S2, and the histogram of their related *I*_p_ and *c* is shown in Figure S8), which also contains the use of traditional methods to detect the recoveries of TDF products, and the use of electrochemical methods to detect the recoveries of TDF and TFV (the key intermediate of TDF [70]). Compared with the recovery of TDF tablets determined by RP-HPLC ((99.57% ± 0.09)~(101.42% ± 0.08)) [17], the recovery of TDF tablets determined by UV-vis spectrophotometry (99.45%~100.4%) [14] and the recovery of TDF tablets determined by AdSDPV (100.35%) and AdSSWV (100.50%) [29], the serum recovery (96.13%~99.70%) and urine recovery (95.87%~101.0%) measured by ZrO_2_-CS-MWCNTs/GCE were consistent. Meanwhile, the recoveries of TFV in the electrochemically detected drugs, using AdSSWV (101.33%) and SWV (95.8% or 101.9%) were also consistent with those in this work. The results show that the electrode can be used for the determination of TDF in actual samples, which is similar to the results obtained from the intermediates and products, and has good feasibility.

## 4. Conclusions

In this work, cubic zirconium oxide was synthesized using the hydrothermal method, and based on this, a new fast and ultrasensitive electrochemical method for the determination of TDF, based on ZrO_2_-CS-MWCNTs/GCE, was studied. The prepared ZrO_2_-CS-MWCNTs composites were characterized using XRD, SEM and EDS. The results showed that under the optimum experimental conditions, ZrO_2_-CS-MWCNTs composites have good electrochemical activity for TDF determination. The ranges of 0.3 μM~30 μM and 0.3 μM~100 μM show good linear correlation with the oxidation current of TDF, and the detection limit reaches 0.0625 μM. In addition, ZrO_2_-CS-MWCNTs/GCE has good selectivity, repeatability, reproducibility and stability for TDF. The sensor can be used in the determination of TDF in serum and urine, with good recovery. The electrochemical sensor is a simple and fast method for TDF detection.

## Figures and Tables

**Table 1:** Comparison of various Tenofovir disoproxil fumarate (TDF) determination methods.

Method	Material	Linear Range (μM)	Detection Limit (μM)	Reference
Spectrophotometry	Acid triphenylmethane dye	6.29~62.9	0.692	[11]
Ultraviolet spectrophotometry	/	7.87~62.9	0.804	[15]
^a^ RP-HPLC	/	236~708	4.75	[67]
First order derivative spectrophotometry	/	7.87~62.9	1.03	[15]
^b^ AdSDPV	GCE	0.6~60	0.102	[29]
^c^ AdSSWV	GCE	0.6~60	0.0839	[29]
^d^ DPV	ZrO_2_-CS-MWCNTs/GCE	0.3~30; 30~100	0.0625	This work

^a^ RP-HPLC: reversed-phase high performance liquid chromatogram. ^b^ AdSDPV: adsorptive stripping differential pulse voltammetry. ^c^ AdSSWV: adsorptive stripping square wave voltammetry. ^d^ DPV: differential pulse voltammetry.

**Table 2:** Recovery of TDF in actual samples.

Sample	Method	Measured/μM	Added/μM	Detected/μM	RSD/%	Recovery/%	Reference
TDF tablets	^a^ RP-HPLC	3.147	1.574	4.743	0.17	101.42 ± 0.08	[17]
		3.147	3.147	6.323	0.25	100.89 ± 0.07	
		3.147	4.721	7.849	0.31	99.57 ± 0.09	
TDF tablets	^b^ UV-Vis	9.441	4.721	/	0.54	100.21	[14]
		9.441	9.441	/	0.94	100.4	
		9.441	14.16	/	0.54	99.45	
TDF tables	^c^ AdSDPV	/	/	/	1.69	100.35	[29]
	^d^ AdSSWV	/	/	/	1.50	100.50	
TFV in pharmaceutical dosage form	AdSSWV	/	/	/	1.36	101.33	[65]
TFV in Viread	^e^ SWV	/	/	/	/	95.8	[71]
TFV in Tenofovir disoproxil Teva		/	/	/	/	101.9	
serum 1	^f^ DPV	^g^ ND	5	4.881	2.91	97.62	This work
		ND	10	9.689	2.38	96.89	
		ND	15	14.42	4.84	96.13	
serum 2		ND	5	4.985	2.01	99.70	
		ND	10	9.810	4.58	98.10	
		ND	15	14.68	2.42	97.87	
urine 1		ND	5	4.862	1.32	97.24	
		ND	10	9.587	2.59	95.87	
		ND	15	14.55	4.96	97.00	
urine 2		ND	5	4.851	0.196	97.02	
		ND	10	9.791	2.11	97.91	
		ND	15	15.15	1.21	101.0	

^a^ RP-HPLC: reversed-phase high performance liquid chromatogram. ^b^ UV-Vis: ultraviolet and visible spectrophotometry. ^c^ AdSDPV: adsorptive stripping differential pulse voltammetry. ^d^ AdSSWV: adsorptive stripping square wave voltammetry. ^e^ SWV: square wave voltammetry. ^f^ DPV: differential pulse voltammetry. ^g^ ND: not detected.

## Data Availability

Not applicable.

## Supplementary Materials

The following supporting information can be downloaded at: https://www.mdpi.com/article/10.3390/bios12121123/s1, Figure S1: Scheme of ZrO_2_-CS-MWCNTs/GCE preparation and detection of Tenofovir disoproxil fumarate (TDF); Figure S2: The energy dispersion spectrometer (EDS) spectrum of ZrO_2_-CS-MWCNTs composites (a); Element mappings of carbon, nitrogen, oxygen and zirconium (c–f) in designated area (b) of ZrO_2_-CS-MWCNTs composites; Figure S3: Differential pulse voltammetry (DPV) diagrams of 10^–4^ M TDF on different electrodes (a: GCE, b: ZrO_2_/GCE, c: CS-MWCNTs/GCE, d: ZrO_2_-CS-MWCNTs/GCE); Figure S4: The possible oxidation mechanism of TDF; Figure S5: Effects of deposition time (a), deposition potential (b) and dropping amount (c) on oxidation peak current of 10^–4^ M TDF on ZrO_2_-CS-MWCNTs/GCE; Figure S6: Inorganic (a) and organic (b) interference experiments of TDF detected by ZrO_2_-CS-MWCNTs/GCE; Figure S7: Repeatability (a), reproducibility (b) and stability (c) of ZrO_2_-CS-MWCNTs/GCE; Figure S8: Peak current of TDF with different concentrations in the actual sample (n = 3); Table S1: Specific peak current of TDF concentration range 0.3 μM~100 μM; Table S2: Recovery of TDF and peak currents of experiments in actual samples (n = 3).
