# Supplementary material for: Fast and Ultrasensitive Electrochemical Detection for Antiviral Drug Tenofovir Disoproxil Fumarate in Biological Matrices
Source: Biosensors (Basel). 2022 Dec 3;12(12):1123. doi: 10.3390/bios12121123 (PMC9775179; doi:10.3390/bios12121123)
Supplement: Supplementary file 1 [file biosensors-12-01123-s001.zip › biosensors-1993382-supplementary.pdf]

Supplementary

Fast and Ultrasensitive Electrochemical Detection for Antiviral Drug Tenofovir Disoproxil Fumarate in Biological Matrices

Jingyun Xiao [1,2,3], Shuting Shi [1], Liangyuan Yao [2,*], Jinxia Feng [1], Jinsong Zuo [1], and Quanguo He [1,2,3,*]

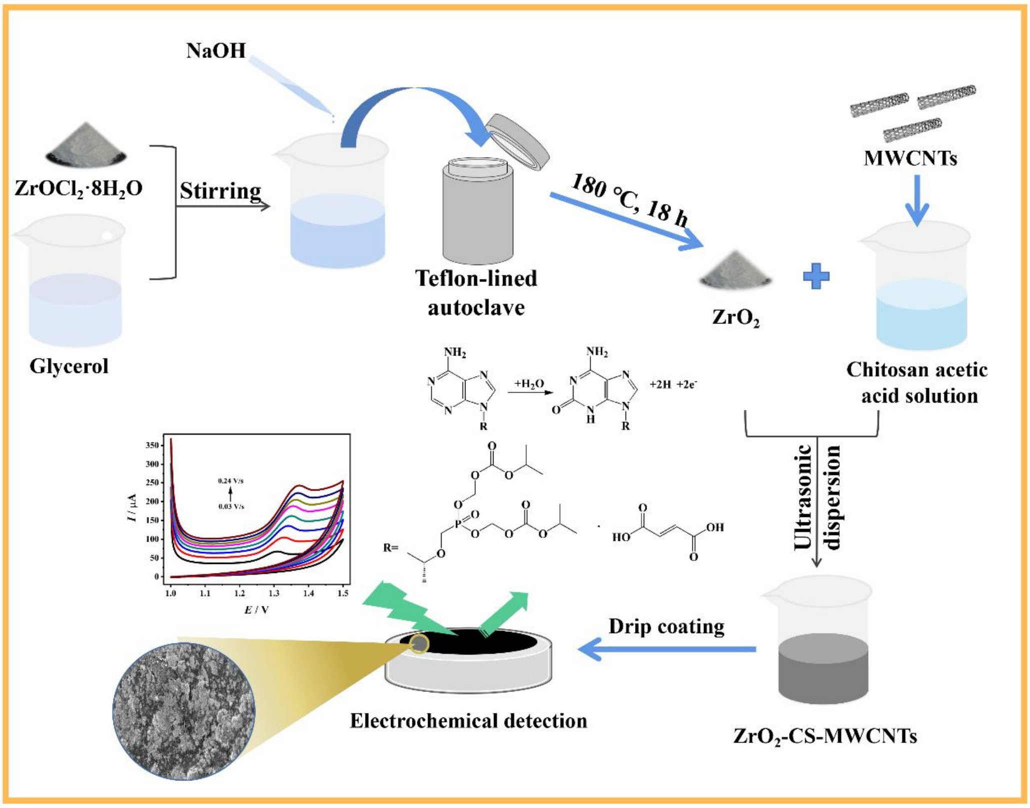

Figure S1. Scheme of ZrO_2-CS-MWCNTs/GCE preparation and detection of Tenofovir disoproxil fumarate (TDF).

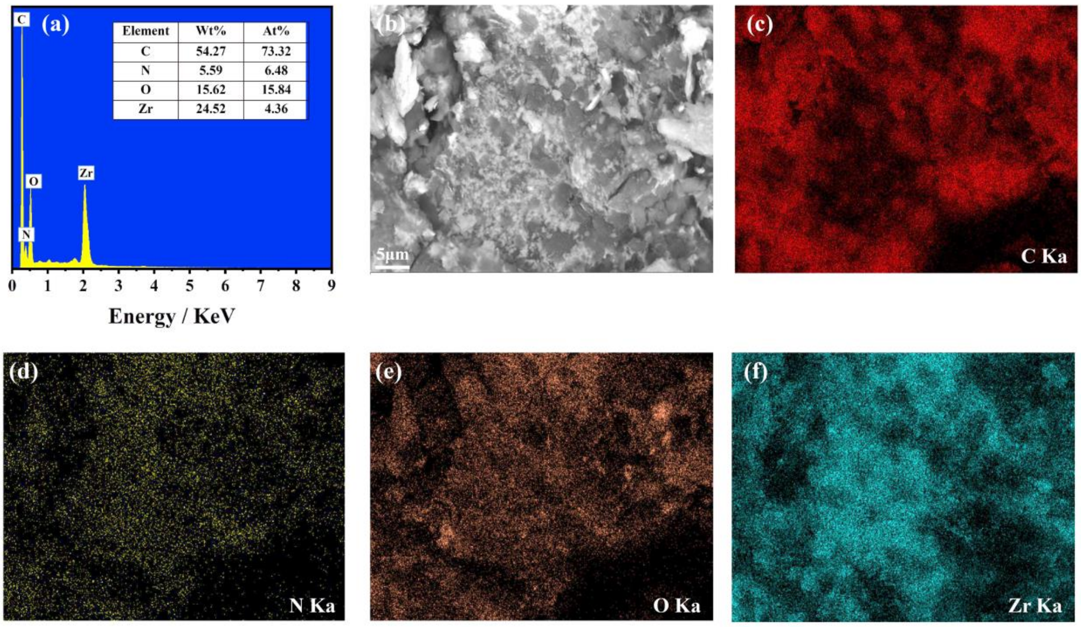

Figure S2. The energy dispersion spectrometer (EDS) spectrum of ZrO_2-CS-MWCNTs composites (a); Element mappings of carbon, nitrogen, oxygen and zirconium (c–f) in designated area (b) of ZrO_2-CS-MWCNTs composites.

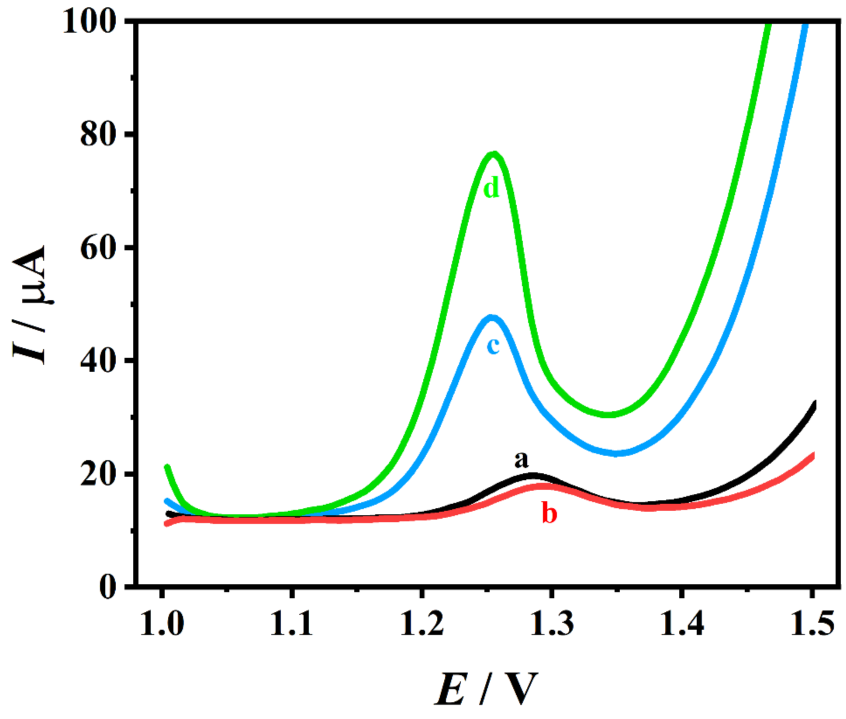

Figure S3. Differential pulse voltammetry (DPV) diagrams of 10^{-4} M TDF on different electrodes (a: GCE, b: ZrO_2/GCE, c: CS-MWCNTs/GCE, d: ZrO_2-CS-MWCNTs/GCE).

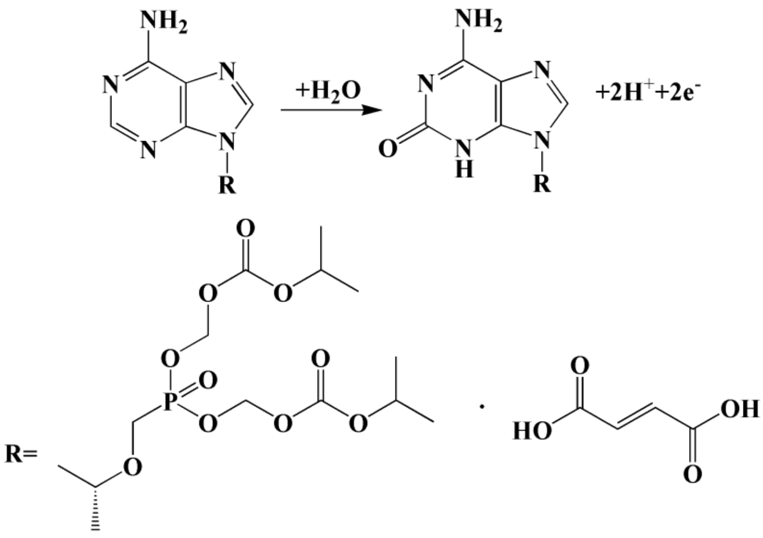

Figure S4. The possible oxidation mechanism of TDF.

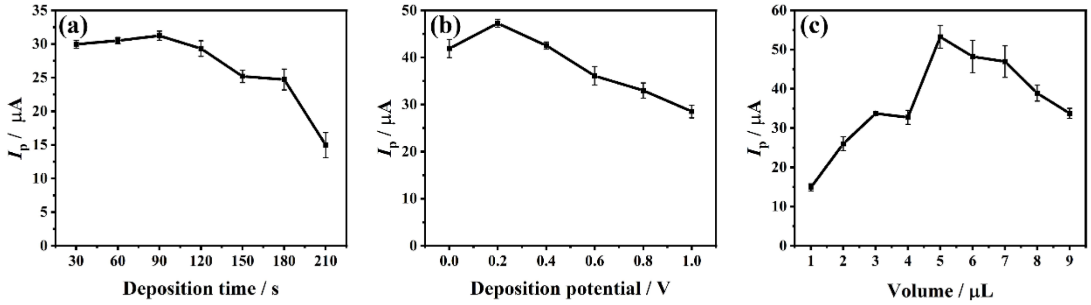

Figure S5. Effects of deposition time (a), deposition potential (b) and dropping amount (c) on oxidation peak current of 10^{-4} M TDF on ZrO_2-CS-MWCNTs/GCE.

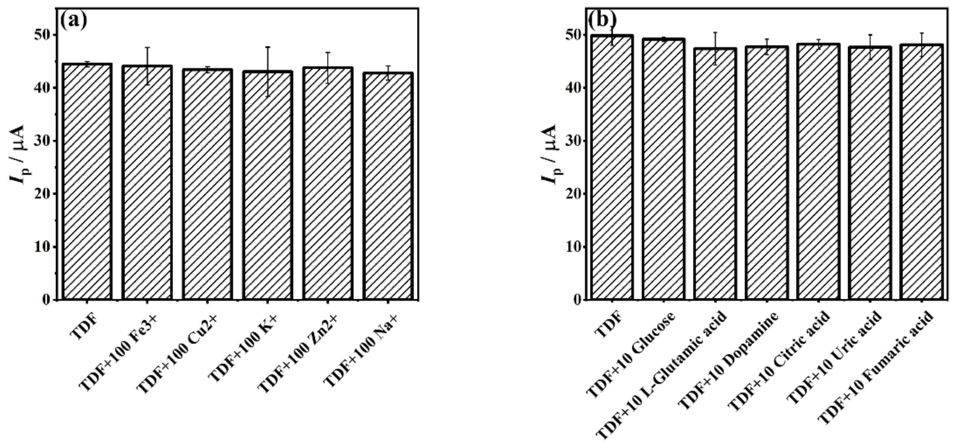

Figure S6. Inorganic (a) and organic (b) interference experiments of TDF detected by ZrO_2-CS-MWCNTs/GCE.

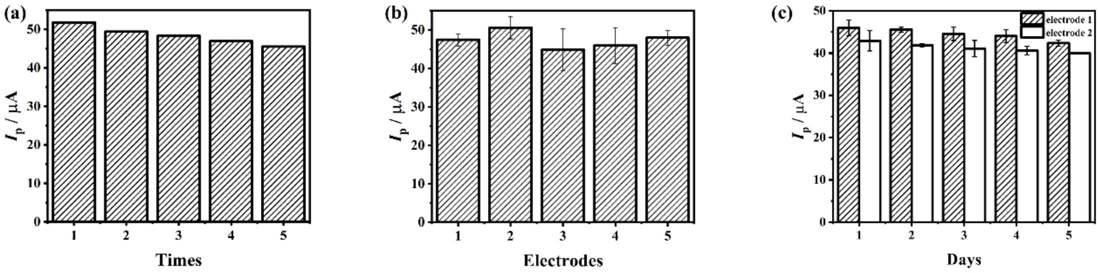

Figure S7. Repeatability (a), reproducibility (b) and stability (c) of ZrO_2-CS-MWCNTs/GCE.

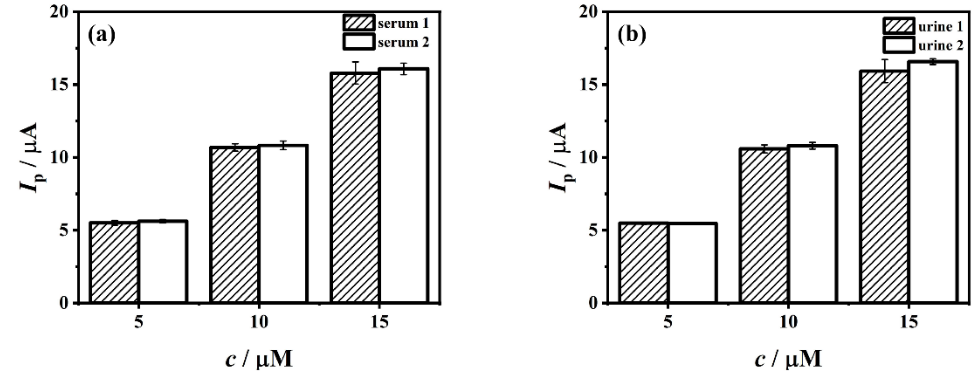

Figure S8. Peak current of TDF with different concentrations in the actual sample (n = 3).

Table S1. Specific peak current of TDF concentration range 0.3 μM ~ 100 μM

c / μM	I_{p1} / μA	I_{p2} / μA	I_{p3} / μA	Mean / μA	Standard Deviation
0.3	0.6002	0.7149	0.6385	0.6512	0.05840
0.5	0.6778	0.7307	0.7354	0.7146	0.03199
0.7	0.9950	0.9858	1.035	1.005	0.02616
0.9	1.213	1.166	1.127	1.169	0.04306
1	1.574	1.813	1.739	1.709	0.1224
3	3.468	2.443	2.846	2.919	0.5164
5	4.831	5.226	4.374	4.810	0.4264
7	7.067	6.637	9.013	7.572	1.266
9	10.30	10.26	10.65	10.40	0.2146
10	11.24	11.84	11.74	11.61	0.3215
30	29.76	29.51	31.01	30.09	0.8036
50	35.16	39.12	36.94	37.07	1.983
70	41.68	45.70	43.93	43.77	2.015
90	49.39	47.39	48.60	48.46	1.007
100	51.76	49.43	48.33	49.84	1.751

Table S2. Recovery of TDF and peak currents of experiments in actual samples (n = 3).

Sample	Measured value / μM	Addition amount / μM	Peak current / μA				RSD / %	Determination of total amount / μM	Recovery rate / %
			first	second	third	average			
serum 1	[a]ND	5.0	5.465	5.678	5.364	5.502	2.91	4.881	97.62
	ND	10.0	10.91	10.74	10.41	10.69	2.38	9.689	96.89
	ND	15.0	15.53	16.65	15.19	15.79	4.84	14.42	96.13
serum 2	ND	5.0	5.566	5.743	5.534	5.614	2.01	4.985	99.70
	ND	10.0	10.61	10.70	11.16	10.82	4.58	9.810	98.10
	ND	15.0	16.18	16.41	15.65	16.08	2.42	14.68	97.87
urine 1	ND	5.0	5.563	5.451	5.428	5.481	1.32	4.862	97.24
	ND	10.0	10.37	10.89	10.48	10.58	2.59	9.587	95.87
	ND	15.0	15.32	16.82	15.64	15.93	4.96	14.55	97.00
urine 2	ND	5.0	5.479	5.458	5.472	5.470	0.196	4.851	97.02
	ND	10.0	10.99	10.55	10.87	10.80	2.11	9.791	97.91
	ND	15.0	16.78	16.38	16.57	16.58	1.21	15.15	101.0

[a]ND = Not detected.
